# Multi-branch CNN and grouping cascade attention for medical image classification

**DOI:** 10.1038/s41598-024-64982-w

**Published:** 2024-07-01

**Authors:** Shiwei Liu, Wenwen Yue, Zhiqing Guo, Liejun Wang

**Affiliations:** School of Computer Science and Technology, Xinjiang University, Urumqi, 830017 Xinjiang China

**Keywords:** Classification and taxonomy, Image processing, Machine learning

## Abstract

Visual Transformers(ViT) have made remarkable achievements in the field of medical image analysis. However, ViT-based methods have poor classification results on some small-scale medical image classification datasets. Meanwhile, many ViT-based models sacrifice computational cost for superior performance, which is a great challenge in practical clinical applications. In this paper, we propose an efficient medical image classification network based on an alternating mixture of CNN and Transformer tandem, which is called Eff-CTNet. Specifically, the existing ViT-based method still mainly relies on multi-head self-attention (MHSA). Among them, the attention maps of MHSA are highly similar, which leads to computational redundancy. Therefore, we propose a group cascade attention (GCA) module to split the feature maps, which are provided to different attention heads to further improves the diversity of attention and reduce the computational cost. In addition, we propose an efficient CNN (EC) module to enhance the ability of the model and extract the local detail information in medical images. Finally, we connect them and design an efficient hybrid medical image classification network, namely Eff-CTNet. Extensive experimental results show that our Eff-CTNet achieves advanced classification performance with less computational cost on three public medical image classification datasets.

## Introduction

Breast cancer, pneumonia, and colon cancer are all common diseases. These diseases seriously jeopardize the health of patients. Rapid and accurate diagnosis of diseases can lead to early prevention and treatment, which is crucial for patients’ health. Medical image classification technology can help doctors quickly identify potential lesions by categorizing medical images, thus improving the accuracy of early diagnosis. However, traditional medical image classification methods face many challenges when dealing with complex medical images, especially there are limitations in terms of sensitivity to smaller focal areas and specific lesions. With the rise of deep learning, especially the application of convolutional neural networks (CNN), medical image classification has entered a whole new era.

CNN are known for their excellent feature learning capabilities and understanding of image hierarchies. Convolutional operations establish a strong connection between a pixel point in an image and the surrounding pixel points, which enables CNN-based network architectures (ConvNet) to effectively capture local detail information. This is crucial for recognizing critical information such as lesions and organ structures, providing strong support for medical image classification tasks. Meanwhile, CNN-based methods do not require excessive training data to achieve better performance. Therefore, CNN-based methods^1,2^ have achieved remarkable success in tasks such as medical image classification and segmentation. However, CNN-based methods are also limited to a fixed-size receptive field, which may restrict the ability of CNN in global information capture, leading to unsatisfactory results in medical images with large differences in texture, shape, and size.

**Figure 1:**
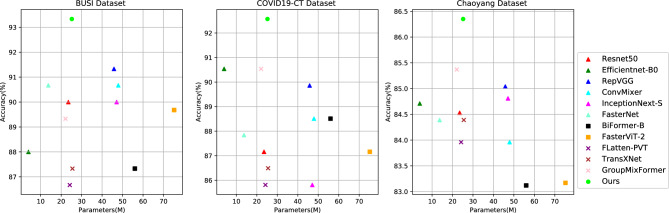
Eff-CTNet and comparison methods in terms of Acc-parameters trade-offs over three datasets.

In recent years, Visual Transformer (ViT)^3^ has significantly improved performance in major visual tasks such as image classification^4^, object detection^5^, semantic segmentation^6^, etc. The ViT model mainly consists of self-attention layer (token mixer) and multi-layer perceptron (MLP) layer (channel mixer). The self-attention mechanism dynamically generates affinity matrices by calculating the similarity between Query and Key, enabling it to establish global dependencies across the entire image range without being limited by the size of the convolutional kernel as in the case of CNN-based methods. However, the increasing performance comes at the cost of increasing model and computational overhead. On the one hand, the computation of global affinity matrices in self-attention is characterized by quadratic complexity and high memory consumption. As a result, these complexity-intensive models face significant challenges in practical clinical applications. To alleviate the computational and memory burden caused by the inherent secondary complexity of self-attention mechanisms, some studies have proposed sparse attention mechanisms. One representative approach is localized attention^7^, which restricts attention to a window on the feature map. However, due to the limited receptive field, this method often requires alternating stacks with different types of token mixers for cross-window information exchange. Another representative approach is to spatially downsample the keys and values of the attention, which sacrifices Query’s fine-grained perception of the feature map, and thus also has some limitations. On the other hand, the key module of the ViT approach that contributes significantly to the performance improvement is multi-head self-attention (MHSA). However, attention maps are computationally expensive and studies have shown that many of these feature sequences are not critical^8,9^. To save computational cost, we explore how to reduce attention redundancy in the ViT model. Meanwhile, although the Transformer model shows a lot of potential in the field of medical images, it is not as good as CNN in extracting local information and requires a large amount of training data, so it is not effective on some small-scale medical image datasets with a small percentage of lesion regions. Therefore, how to design the network architecture to focus well on local features while effectively establishing remote dependencies has triggered our thinking.

In this paper, in order to reduce the computational cost of MHSA operations and reduce redundancy. We propose an efficient Transformer (ET) module. The core of the ET module is group cascade attention (GCA). In order to explicitly encourage the heads in MHSA to learn different patterns, the GCA module divides the feature map into groups, i.e., only a portion of the feature map is provided to each head (inspired by the group conv in literature^10^), thus explicitly disaggregating the attention computation of individual heads. However, we still want the module to learn richer feature information, so we compute the attention graph for each head in a cascading manner. This operation further improves the attentional diversity while effectively reducing the redundancy of attentional computation. In addition, due to the problem of a small percentage of lesion regions and the lack of a large amount of training data in medical images. In order to enhance the network’s learning of local detail information, we propose an efficient CNN (EC) module, which employs a multi-branch structure to learn richer local feature information. Finally, we design a new efficient medical image classification network (Eff-CTNet) by alternating the EC and ET module stages in series and optimizing the network complexity. Each stage of Eff-CTNet consists of two basic building blocks, EC and ET, in tandem, which focus on the local detail information in medical images and at the same time effectively focus on the global information, thus improving the classification performance of the network. Finally, we conducted extensive experiments on three public medical image classification datasets. The experimental results show that our proposed Eff-CTNet achieves better classification results than existing methods based on CNN, ViT, and their hybrid methods with a small computational expenditure. As shown in Fig. 1, our Eff-CTM achieves a better trade-off between the number of model parameters and classification accuracy two small and one larger public medical image datasets.

In summary, the contributions of this paper are as follows:

1. We propose the ET module and the GCA module. The GCA module divides the feature maps into different groups, i.e., only a part of the feature maps is provided to each head, while another chunk is computed inside each head, followed by the computation of the attention maps in a cascading manner, which effectively mitigates the redundancy of the attentional computation while further improving the attentional diversity.
2. We propose the EC module, which employs a multi-branch CNN structure to learn richer local feature information. The structure of the EC module is also optimized to further reduce the number of parameters and FLOPs of the model.
3. We cascade the EC module and ET module level alternately in series and use this as a base building block to design an efficient medical image classification network, Eff-CTNet, and we have conducted extensive experiments on three public medical image classification datasets. The experimental results show that our Eff-CTNet achieves state-of-the-art classification performance with less number of parameters and FLOPs.

## Related work

### CNN-based methods

CNN have dominated the field of image classification in the last decade. CNN have been widely used and intensively studied since the advent of AlexNet^11^. ResNet^12^ introduced residual connection, which allowed deep networks to become as easy to train and optimize as shallow networks. This design concept has had a profound impact on many subsequent models, giving rise to numerous improved and variant models. RepVGG^13^ uses a structural reparameterization technique, which employs a multi-branch topology during the training process and a single-branch structure similar to that of VGG^14^ during the inference phase. This design allows the model to have higher speed, lower memory consumption, and better flexibility. RepLKNet^15^ also employs a structure-heavy parameterization technique and uses deep convolution and a very large 31×31 convolution kernel. This structure is fast and performs well, but the model is larger. ConvNext^16^ is influenced by Swin Transformer^17^, which optimizes the structure, training strategy, and data augmentation techniques of ResNet50 to improve the performance of the model. However, it requires a large amount of data. During this period, some other lightweight methods have been proposed. Literature^18,19^ are all classical lightweight networks designed to run on mobile and embedded devices. Recently, FasterNet^20^ proposed a novel operator called partial convolution, which can extract spatial features more efficiently and faster. InceptionNext^21^ combines Inception^22^ with the ConvNext model and excels in both performance and practical efficiency. Similarly, CNN are widely used in medical image classification tasks. DermoExpert^23^ used a preprocessing approach and combined a hybrid CNN with three different feature extractor modules to achieve the classification of skin diseases. ResGANet^24^ proposed a modularized group attention block to capture key features in medical images in spatial and channel dimensions, respectively, to improve classification performance. Literature^25^ proposed a spiking cortical model based global and local (SCM-GL) attention module, thus effectively improving the classification performance of lightweight CNN methods.

### ViT-based methods

ViT^3^ applied Transformer to vision for the first time and achieved impactful results. CrossViT^26^ proposed a two-branch Transformer to integrate image tokens of different sizes to extract better feature information. BiFormer^27^ addressed the original Transformer architecture problem of high computational cost, proposed a dynamic sparse attention that achieves more flexible computational allocation and content awareness. GroupMixFormer^28^ proposed group-mix attention (GMA), which simultaneously captures token-to-token, token-to-group, and group-to-group correlations for different group sizes. Flattern Transformer^29^ analyzed the shortcomings of existing linear attention methods and proposed a plug-and-play focused linear attention (FLA) module with both high efficiency and strong model representation. In recent years, ViT have also been applied to medical image classification tasks. Pocformer^30^ proposed a lightweight Transformer model for the diagnosis of neocoronary pneumonia. RadioTransformer^31^ proposed a novel student-instructor Transformer framework and on a dataset of eight different disease classifications validated its effectiveness. In addition, RMT-Net^32^ used Transformer to capture long-range feature information and convolutional neural network and deep convolution to obtain local features for COVID-19 detection.

### Hybrid methods

Conformer^33^ is the first hybrid network that combines CNN and Transformer in parallel, the feature coupling unit (FCU) achieves the interaction of local and global features at various stages, harnessing the advantages of both. Next-ViT^34^ constructs an efficient deployment model suitable for real-world industrial scenarios by stacking and blending CNN and Transformer modules. TransXNet^29^ introduces a novel hybrid network module, dual dynamic token mixer (D-Mixer), which aggregates global information and local details in a manner dependent on the input, effectively expanding the network’s receptive field. Transmed^4^ applies ViT to medical image classification tasks for the first time, utilizing a hybrid structure based on CNN and Transformer for the classification of parotid tumors in multimodal medical images. MedViT^35^ proposes a highly robust and effective hybrid model combining CNN and Transformer, demonstrating high robustness and generalization on large-scale standardized medical datasets with relatively low computational complexity. CVM-Cervix^36^ presents a hybrid model integrating CNN, Transformer, and MLP for cervical pap smear image classification.

**Figure 2:**
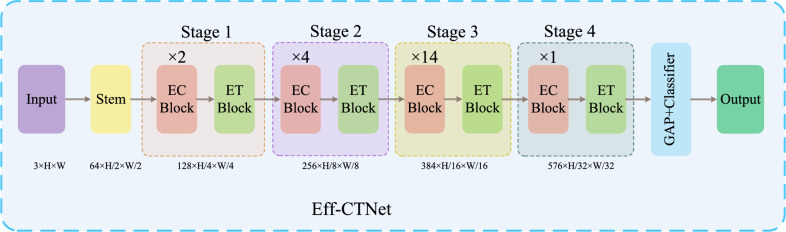
Overview of Eff-CTNet. Eff-CTNet consists of EC and ET Block.

## Method

### Eff-CTNet

The overall network architecture of Eff-CTNet is shown in Fig. 2, with four stages. Previously, many hybrid methods based on CNN and Transformer have used CNN structures in the shallow layers of the network to extract local information, followed by Transformer structures in the deeper layers of the network to extract global information. However, since the lesion region in medical images accounts for a relatively small area, and the lesion morphology is affected by many factors such as different patient’s physiques. Therefore, the above design method may lead to inadequate extraction of feature information in medical images. To further extract richer local and global information in medical images, we set Eff-CTNet to consist of an efficient CNN (EC) module and an efficient Transformer (ET) module in series in each stage. The input image of Eff-CTNet ${X_{in}}\in \textrm{R}^{3 \times H \times W}$, which is first downsampled in the stem layer by 3$\times 3$ group conv with a step size of 2. The height and width of the feature map are each reduced by half, and the number of channels is increased to 64 to output the feature map ${F_{1}}\in \textrm{R}^{64 \times \frac{H}{2} \times \frac{W}{2}}$ . The design of the stem layer effectively reduces the input size and parameters of the model. Next, the output feature maps after executing the EC module and ET module 2 times and 4 times in stage1 and 2, respectively are ${F_{2}}\in \textrm{R}^{128 \times \frac{H}{4} \times \frac{W}{4}}$ and ${F_{3}}\in \textrm{R}^{256 \times \frac{H}{8} \times \frac{W}{8}}$. Our Eff-CTNet maintains the same design principle as successful architectures such as RepVGG^13^, ResNet^12^, etc., by setting the most layers of the network at the penultimate stage of the network (stage 3). However, as the depth of the network increases, if the number of channels in the last two stages (stage 3, 4) is doubled in the same way as in the first two stages (stage 1, 2), the complexity of the whole network will also increase dramatically. Therefore, in order to reduce the number of parameters and FLOPs of the model, we set the number of channels in stage 3, 4 to 1.5 times of the previous stage. Meanwhile the number of repetitions of the EC module in stage3, 4 is reduced to 14 and 1. The final output feature maps are respectively ${F_{4}}\in \textrm{R}^{384 \times \frac{H}{16} \times \frac{W}{16}}$ and ${F_{5}}\in \textrm{R}^{576 \times \frac{H}{32} \times \frac{W}{32}}$ . Then global average pooling is applied to the feature map ${F_{5}}$ , which is finally fed into a full connection layer as a classification head to complete the disease classification.

### EC module

**Figure 3:**
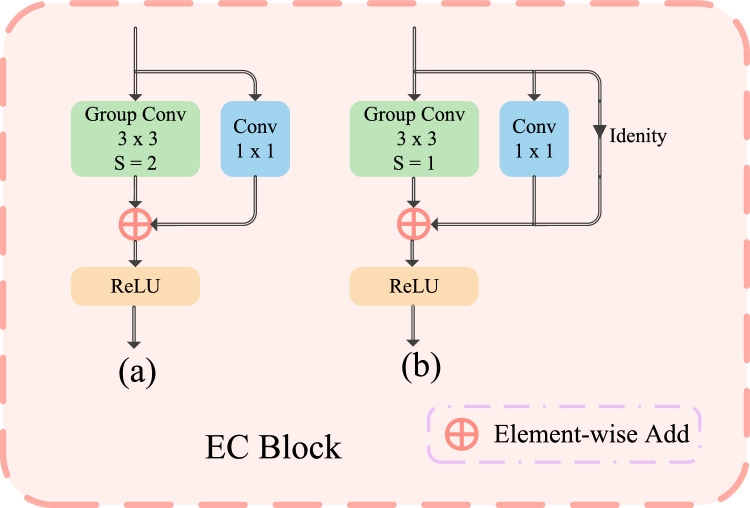
Example of EC Block structure. (**a**) is the EC block including downsampling, (**b**) is the EC Block without downsampling.

The EC module is used in each stage of Eff-CTNet, and its specific structure is shown in Fig. 3. The EC module is similar to the basic building blocks in the training process in the baseline^13^, and we improve it by retaining the multi-branch topology, which exhibits a more powerful characterization capability. Meanwhile, to reduce the number of parameters and computational complexity of the model, we replace the original conventional conv with group conv. The EC module has two structures, as shown in Fig. 3 (a) and Fig. 3 (b). Fig. 3 (a) represents the structure with downsampling, where each convolution block consists of a step size of 2 of the 3×3 group conv and 1×1 conv branches. Then the two branches are summed through ReLU^37^ to get the final output. While Fig. 3 (b) represents the structure without downsampling, each convolutional block consists of a step size of 2 of the 3×3 group conv, 1×1 conv, and identity branches, and again the results of the three branches are summed up before going through the ReLU activation function to get the final output. In stages 1, 2, 3, and 4, the first EC module uses the structure of Fig. 3 (a), and each subsequent EC module uses the structure of Fig. 3 (b).

### ET module

**Figure 4:**
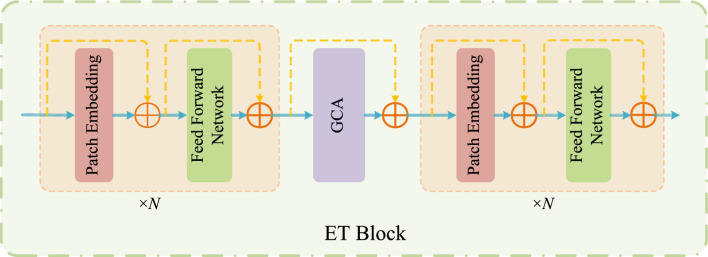
Specific structure of the ET module in Eff-CTNet.

In order to allow the network to better learn the remote dependencies in medical images, we propose an efficient Transformer (ET) module. The ET module is one of the core building blocks in each stage of Eff-CTNet, and its structure is shown in Fig. 4. The sandwich-style layout has been shown by literature^38^ to effectively improve the memory efficiency of the model. Therefore, we are inspired by and propose the ET module with a sandwich-style layout, which is mainly communicated by the patch embedding layer, the efficient feed forward network (FFN) layer, and the grouped cascade attention (GCA) module. Among them, the patch embedding layer is also realized by 3$\times$3 group conv and the FFN layer is realized by 1$\times$1 convolution. Such a design strategy helps to improve the efficiency of the model in terms of computational spend and parameters. Specifically, the ET module applies a single self-attention layer $\Phi _{i}^{\textrm{A}}$ for spatial information mixing, which is sandwiched between two FFN layers $\Phi _{i}^{\textrm{F}}$. The ET module is designed for spatial information mixing. The exact working principle can be described as follows:

1 $$\begin{aligned} T_{i+1}=\prod ^{\mathcal {N}} \Phi _{i}^{\textrm{F}}\left( \Phi _{i}^{\textrm{A}}\left( \prod ^{\mathcal {N}} \Phi _{i}^{\textrm{F}}\left( T_{i}\right) \right) \right) , \end{aligned}$$

where $T_i$ denotes the input feature map of the *i*-th block. The ET module, after using *N* patch embedding and FNN layers before and after a single GCA layer, respectively, will $T_i$ converted to $T_{(i+1)}$. The ET module is designed in such a way that it effectively reduces the computational spend of the self-attention layer and utilizes more FFN layers to fuse the feature information communication of different channels. Meanwhile, we apply a patch embedding layer before each FFN layer, which utilizes deep convolution to introduce an inductive bias of local feature information to further enhance the feature learning capability of the model.

### GCA module

**Figure 5:**
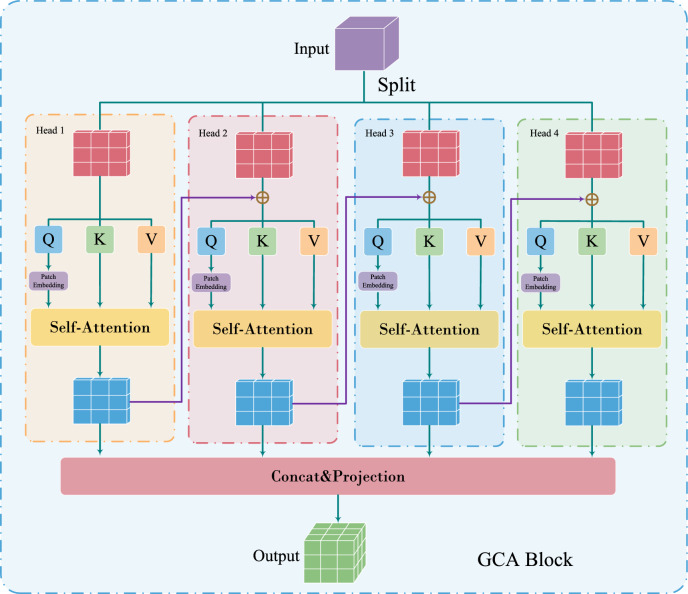
Specific structure of the GCA module in ET module.

The success of ViT^3^ is largely attributed to the self-attention mechanism. Self-attention mechanisms in MHSA embed the input sequences into multiple subspaces (heads) and compute the attention maps separately, which has been shown to be effective in improving performance^3,39^. However, attentional redundancy in MHSA is an important issue that leads to its computational inefficiency. In order to reduce the computational redundancy in MHSA, inspired by group conv^10^ in efficient CNN and literature^38^, we propose a new grouped cascade attention (GCA) module, which is the core of the ET module, and its specific structure is shown in Fig. 5. The GCA module divides the feature map into groups along the channel dimension, i.e., it provides each head with only a feature map part of the feature map to each head (similar to group conv), thus explicitly decomposing the attention computation of each head. Formally, GCA can be formulated as follows:

2 $$\begin{aligned} \begin{aligned}{}&\tilde{X}_{i j} ={\text {Attn}}\left( X_{i j} W_{i j}^{\textrm{Q}}, X_{i j} W_{i j}^{\textrm{K}}, X_{i j} W_{i j}^{\textrm{V}}\right) , \\&\widetilde{X}_{i+1} ={\text {Concat}}\left[ \widetilde{X}_{i j}\right] _{j=1: h} W_{i}^{\textrm{P}}, \end{aligned} \end{aligned}$$

where the *j*-th head computes the self-attention over $X_{ij}$, which is the *j*-th split of the input feature $X_i$, i.e., $X_i$ = [$X_{i1}$,$X_{i2}$, ... , $X_{ih}$] and 1 $\le$ *j* $\le$ *h*. *h* is the total number of heads, $W_{i j}^{\textrm{Q}}$ , $W_{i j}^{\textrm{K}}$ , and $W_{i j}^{\textrm{V}}$ are projection layers mapping the input feature split into different subspaces, and $W_{i}^{\textrm{P}}$ is a linear layer that projects the concatenated output features back to the dimension consistent with the input.

Then, we divide the feature maps in the spatial dimension inside each head into n windows of the same size for self-attention computation respectively, and this design dramatically reduces the computational spend of the model, and its operation principle can be described as follows:

3 $$\begin{aligned} X_{i j}^{\prime }=X_{i j}+\tilde{X}_{i(j-1)}, \quad 1<j \le h, \end{aligned}$$

where $X_{i j}^{\prime }$ is the addition of the *j*-th input split $X_{i j}$ and the (*j*-1)-th head output ${X}_{i(j-1)}$ calculated by Eq. 2. It replaces $X_{i j}$ to serve as the new input feature for the *j*-th head when calculating the self-attention.

Although we use only a portion of the feature segmentation rather than the entire feature map for each head, the former approach is more efficient and saves planning overhead compared to the latter. However, we still want the module to learn richer feature information, so we compute the attention graph for each head in a cascading manner. As shown in Fig. 5, the GCA module sequentially adds the output of the previous head to the latter head for further feature refinement. In addition to this, we apply a Patch Embedding layer after the Q-projection, and doing so allows self-attention to capture both local and global relationships and further enhance the feature representation. This cascade design approach has two advantages. First, it provides a different grouping of features for each head, thus increasing the diversity of the attention graph. Similar to group conv, since the input and output channels of the QKV layer in the GCA are reduced by a factor of *h*, the number of parameters and FLOPs of the GCA are thus saved by a factor of *h*. Second, the depth of the network can be increased by cascading the attention heads, which further enhances the capacity of the model without introducing any additional parameters.

### Loss function

The cross-entropy loss function can measure the difference between two probability distributions and has better performance in the classification task. In the medical image classification task, the output probability distribution of the model and the probability distribution of the real label often have certain differences, and by minimizing the cross-entropy loss, the output probability distribution of the model can be closer to the probability distribution of the real label, to improve the accuracy of classification. At the same time, the cross-entropy loss function has a better gradient property. In the training process, the gradient form of the cross-entropy loss function is better, which helps optimize the model parameters and improve the convergence speed and accuracy of the model. By minimizing the cross-entropy loss function, the model can be made to be gradually optimized during the training process to improve the classification performance. Since medical image classification tasks usually involve multiple categories, such as identifying different lesion types or tissue structures. The cross-entropy loss function performs well in multi-category classification problems. Therefore, cross-entropy loss is used as the loss function in this paper. The computational equation of CrossEntropyLoss is as follows:

4 $$\begin{aligned} \mathcal {L}_{\text{ CrossEntropyLoss }}=-\sum _{x=1}^{N} p(x) \cdot \log (q(x)), \end{aligned}$$

where *N* represents the batch size, *p*(*x*) represents the true label, and *q*(*x*) is the prediction probability.

## Experiments and analysis

### Datasets

In this paper, we conduct extensive experiments on three public medical image datasets to validate the effectiveness of our proposed method.

**Table 1:** Distribution of lesions in the BUSI dataset.

Dataset split	Normal	Benign	Maligant	Total
Train	109	353	168	630
Test	24	84	42	150
Total	133	437	210	780

**Table 2:** Distribution of lesions in the COVID19-CT dataset.

Dataset split	Covid	NonCovid	Total
Train	280	318	598
Test	69	79	148
Total	349	397	746

**Table 3:** Distribution of lesions in the Chaoyang dataset.

Dataset split	Normal	Serrated	Adenocarcinoma	Adenoma	Total
Train	1111	842	1404	664	4021
Test	705	321	840	273	2139
Total	1816	1163	2244	937	6160

**(1) Breast ultrasound images dadaset**:The BUSI dataset was released in 2020 by literature^40^ and contains 780 breast ultrasound images collected from 600 female patients. These images had an average size of 500×500 pixels and were classified into three categories: normal, benign tumors, and malignant masses. There were 133 normal images, 437 benign tumor images, and 210 malignant mass images in the dataset. In the experiments of this paper, we randomly divided the dataset into 630 training samples and 150 test samples according to the ratio of 8:2.The specific data distribution of the BUSI dataset is shown in Table 1.

**(2) COVID19-CT dataset**:The COVID19-CT dataset^41^ is a dichotomous dataset, which has 746 samples. Among them, there are 349 positive samples for new crown pneumonia and 397 negative samples without clinical manifestations of new crown pneumonia. We randomly divided each category of the dataset into a training set and a test set in the ratio of 8:2. There were 598 samples in the training set and 148 samples in the test set. The data distribution of the COVID19-CT dataset is shown in Table 2.

**(3) Chaoyang Dataset**:Chaoyang Dataset^42^ is a Colon slides dataset, which is constructed from real scenes collected from Chaoyang Hospital in Beijing. The dataset contains four categories: normal, serrated, adenocarcinoma, and adenoma, with 6160 samples and a slice size of 512×512. We compared with literature^42^ to maintain a consistent division, 1111 normal, 842 serrated, 1404 adenocarcinoma, 664 adenoma samples for training, and 705 normal, 321 serrated, 840 adenocarcinoma 273 adenoma samples for testing. The distribution of data in the Chaoyang dataset is shown in Table 3.

### Experimental details

In all the experiments in this paper, we used a series of rigorous settings to ensure the reliability and validity of the experiments. First, the image size of the input model for all experiments was set to 224 × 224 by default, with a batch size of 32. For image preprocessing, we only used the basic operations of random cropping, random horizontal flipping, and normalization, and did not perform any other data enhancement techniques beyond that. Second, during model training, we used the Adam^43^ optimizer with a weight decay of 0.1. We set the initial learning rate to 0.0001 and employed a cosine annealing decay strategy to dynamically adjust the learning rate. Finally, we train all models for 300 epochs by default. All experiments in this paper are trained and tested on a single NVIDIA TITAN RTX 24G GPU.

### Evaluation metrics

In the medical image classification task, a single evaluation metric often fails to fully reflect the performance of the model. In order to accurately and reliably evaluate the model performance, four metrics, Accuracy (Acc), Precision, Recall, and F1 score, are chosen to evaluate the classification performance of the model in this paper. Acc is a very important metric in the classification task, which measures the ratio of the number of samples correctly classified by the model to the total number of samples. Meanwhile, Precision and Recall are also commonly used evaluation metrics. Precision measures the proportion of true instances that the model predicts as positive, while Recall measures the ability of the model to correctly predict true instances. However, in some cases, Precision and Recall may be contradictory to each other, so in this paper, we will consider both of them together and use the F1 score as one of the evaluation metrics, which combines Precision and Recall to evaluate the classification performance of the model. In addition, we use the receiver operating characteristic (ROC) curve and the area under the receiver ROC curve (AUC) as evaluation metrics to assess the classification performance of different models. The ROC curve depicts the model’s ability to recognize positive and The AUC measures the area under the ROC curve, which reflects the model’s overall ability to recognize positive and negative examples.

In summary, Accuracy, Precision, Recall, F1 score as well as ROC curve and AUC are selected as evaluation metrics in this paper, which can complement each other to assess the performance of the model in medical image classification tasks from multiple perspectives. The calculation methods of these evaluation indexes are as follows:

5 $$\begin{aligned} Accuracy(Acc)=\frac{TP+TN}{TP+FP+TN+FN}, \end{aligned}$$

6 $$\begin{aligned} Precision =\frac{TP}{TP+FP}, \end{aligned}$$

7 $$\begin{aligned} Recall=\frac{T P}{T P+F N}, \end{aligned}$$

8 $$\begin{aligned} F 1 =\frac{2 * Precision * Recall }{ Precision +Recall }, \end{aligned}$$

where truth positive is the *TP*, false positive is *FP*, true negative is *TN*, and false negative is *FN*. The *AUC* is calculated as follows:

9 $$\begin{aligned} A U C=\frac{\sum _{i \in positiveClass } r a n k_{i}-{M(1+M)}/{2}}{M * N}, \end{aligned}$$

where *M* is the number of positive samples, *N* is the number of negative samples, and $rank_i$ is the rank of the model’s on the prediction probability of sample *i*.

### Experimental results

#### Results of comparison experiments on the BUSI dataset

**Table 4:** Results of comparison experiments on the BUSI dataset.

Method(year)	Params (M)	FLOPs (G)	Acc	F1	Precision	Recall	Auc
ResNet50 (2016)	23.5	4.1	0.9000	0.8769	0.9164	0.8492	0.8909
MobileNetV2 (2018)	2.2	0.3	0.8667	0.8404	0.8849	0.8115	0.8598
EfficientNet-B0 (2019)	4.0	0.4	0.8800	0.8628	0.9063	0.8353	0.8756
RepVGG (2021)	43.7	9.9	0.9133	0.8964	0.9102	0.8869	0.9162
ConvNext-S (2022)	49.5	8.7	0.7667	0.6717	0.8503	0.6290	0.7283
ConvMixer (2023)	47.9	49.1	0.9067	0.8901	0.9006	0.8810	0.9117
InceptionNext-S (2023)	47.1	8.4	0.9000	0.8906	0.9066	0.8770	0.9060
FasterNet (2023)	13.7	1.9	0.9067	0.8907	0.9080	0.8790	0.9122
Swin-S (2021)	48.8	8.6	0.8400	0.8219	0.8515	0.8095	0.8521
CrossViT 18 (2021)	43.3	9.0	0.8600	0.8308	0.8679	0.8155	0.8619
MoCoViT 1.0 (2022)	7.2	0.5	0.8600	0.8373	0.8838	0.8095	0.8563
BiFormer-S (2023)	56.0	9.4	0.8733	0.8493	0.8950	0.8194	0.8653
FasterViT-2 (2023)	75.2	8.9	0.8968	0.9173	0.8867	0.8736	0.8712
Flatten-pvt (2023)	24.2	3.7	0.8667	0.8497	0.8811	0.8333	0.8720
TransxNet (2023)	25.5	4.6	0.8733	0.8576	0.8674	0.8492	0.8861
GroupMixFormer (2023)	22.1	5.1	0.8933	0.8657	0.9285	0.8294	0.8772
Eff-CTNet(Ours)	25.2	6.4	**0.9333**	**0.9261**	**0.9326**	**0.9226**	**0.9404**

Bold indicates the optimal metric values among all compared methods.

**Figure 6:**
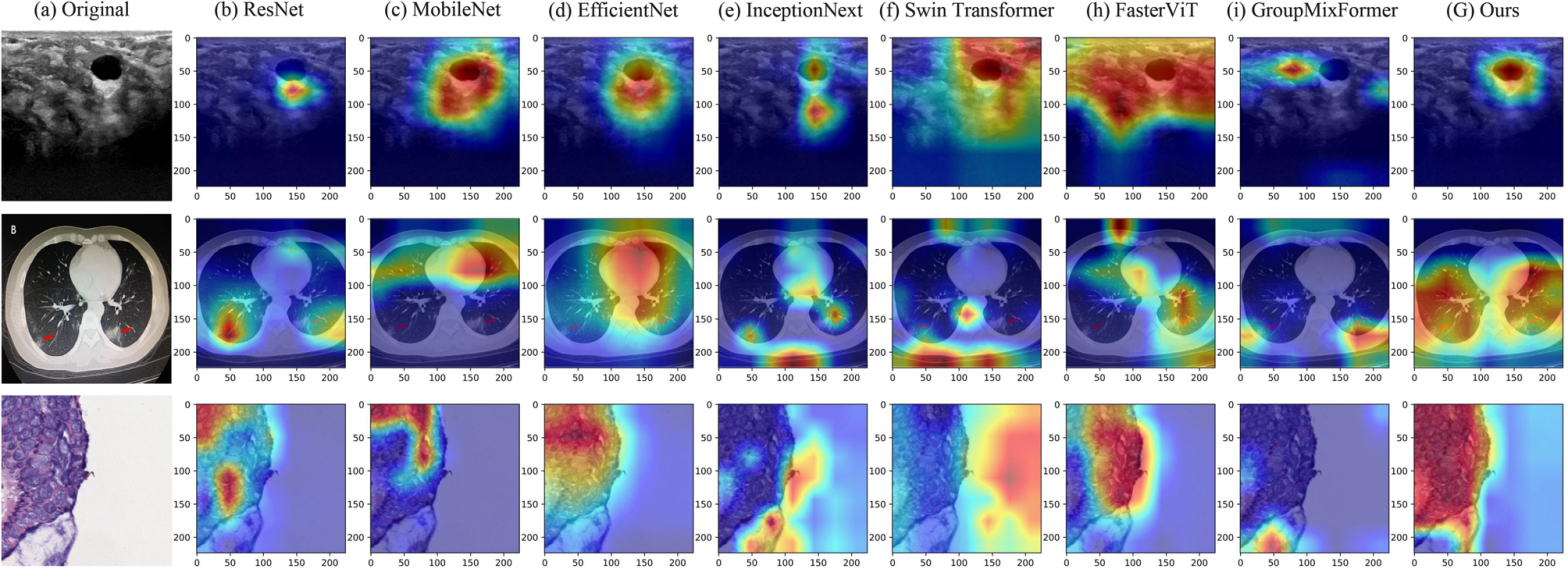
Grad-CAM visualization results for different comparison models on the BUSI, COVID19-CT, Chaoyang datasets.

The results of our experiments comparing Eff-CTNet with other state-of-the-art methods are shown in Table 4. By comparing the five classification metrics in the table, we can clearly observe that the classification performance of the CNN-based methods outperforms the ViT-based methods overall. For example, the classical ResNet50 achieves 90% Acc, 87.69% F1, 91.64% Precision, 84.92% Recall, and 0.8909 AUC, while Swin Transformer achieves only 78% Acc, 72.18% F1, 84.92% Precision, 67.46% Recall, and 0.7574 AUC. In contrast, the former’s classification performance on the BUSI dataset is much better than the latter’s. We analyze the main reasons for this difference. We analyze that the main reason for this difference may be that the BUSI dataset has a small amount of data, and the CNN-based method is able to use convolutional operations to extract local information, while it does not require much training data to achieve better performance. However, the ViT-based method does not perform well on the BUSI dataset, which has a small percentage of lesion regions and a small amount of data, to remove the full performance. It is worth mentioning that our Eff-CTNet achieves 93.33% Acc, 92.61% F1, 93.26% Precision, 92.66% Recall, and 0.9404 AUC on the BUSI dataset, respectively, which outperforms the CNN-based approach in all metrics while the number of parameters and FLOPs are small, Transformer and their hybrid methods. Compared to the baseline (RepVGG), our method improves Acc by 2%, F1 by 2.97%, Precision by 2.24%, Recall by 3.97%, and AUC by 2.42% with only 55% of the latter’s number of parameters and 65% of its FLOPs. Eff-CTNet achieves a substantial improvement in classification performance while reducing complexity. The substantial improvement in classification performance, which validates the effectiveness of our method.

**Figure 7:**
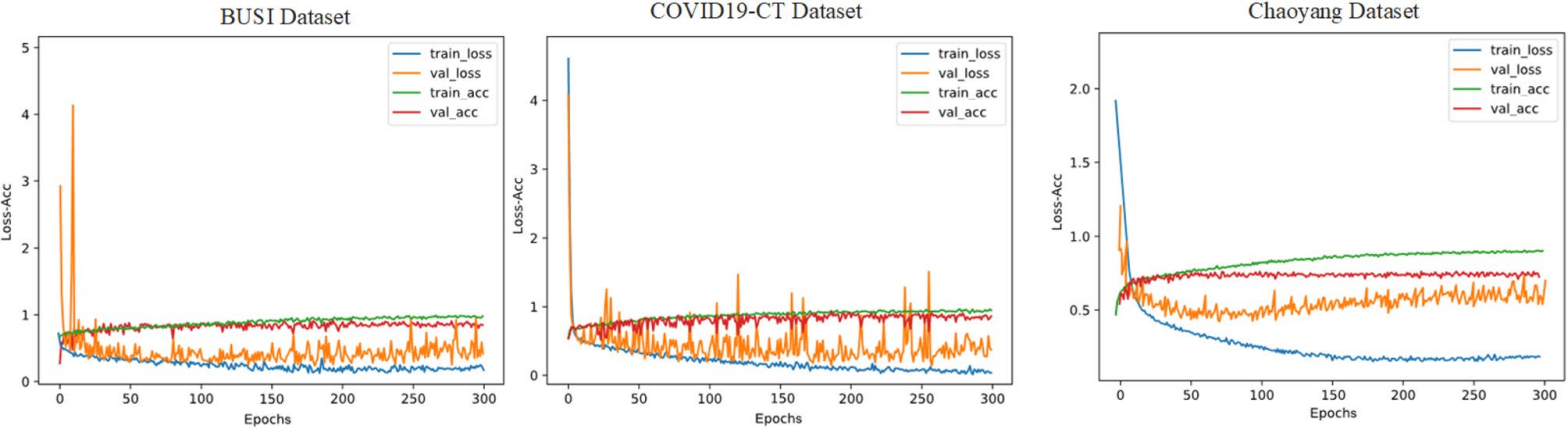
Training curves of Eff-CTNet on BUSI, COVID19-CT and Chaoyang datasets.

**Figure 8:**
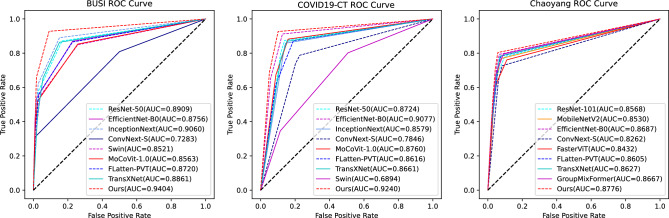
ROC curves for different comparison methods on the BUSI, COVID19-CT, Chaoyang datasets.

**Figure 9:**
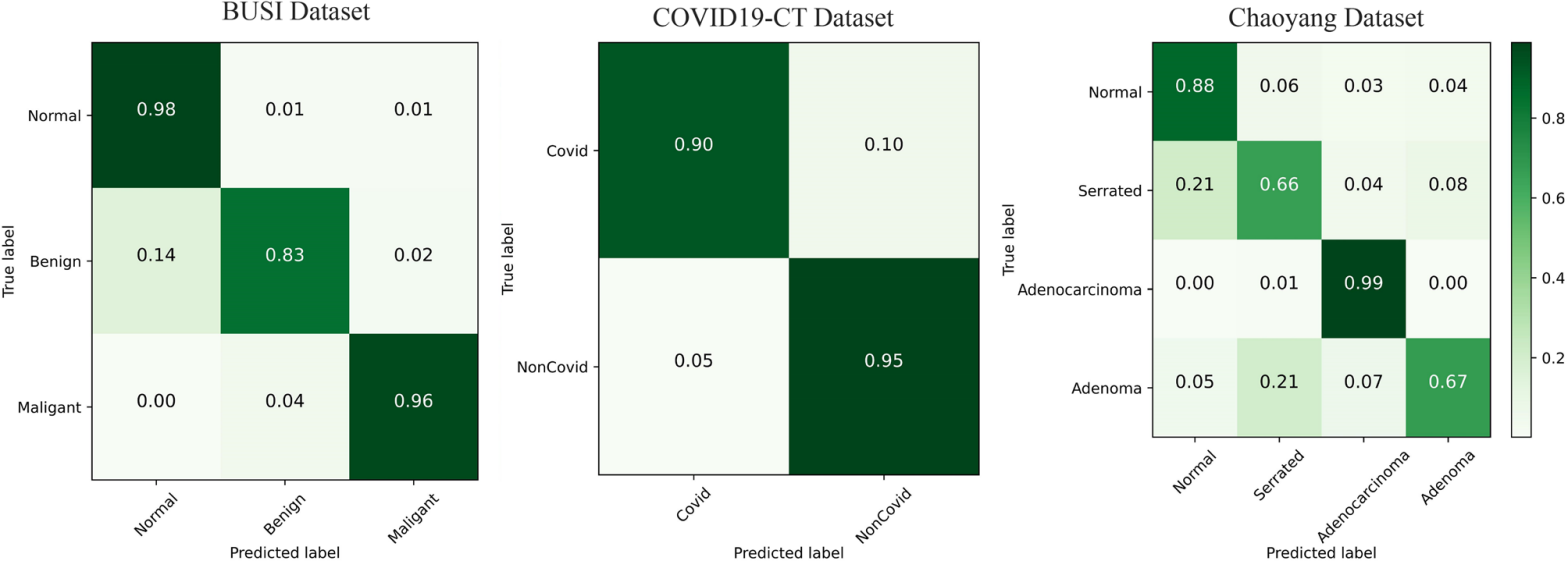
Confusion matrix visualization of Eff-CTNet on the BUSI, COVID19-CT, Chaoyang datasets.

The EC module in Eff-CTNet is able to better focus on local features, while the ET module takes into account local information while focusing on remote dependencies through the CGA operation. Eff-CTNet enables the network to learn richer feature information by connecting the EC and ET modules in tandem. The first row of Fig. 6 shows the Grad-CAM^44^ visualization of benign samples from the BUSI dataset on different methods. By comparing the visualization results in different columns, we notice that the CNN-based method is able to focus on the lesion area better compared to the ViT-based method. While our Eff-CTNet accurately locates the lesion region, the visualization results of Grad-CAM further verify the authenticity of the metrics in Table 4. The left side of Fig. 7 shows the training graph of our method on the BUSI dataset. From the figure, we observe that the model gradually converges as the number of training epochs increases. Meanwhile, the difference between the training loss and accuracy of the model and the validation loss and accuracy is small, which verifies the strong generalization ability and stability of the model. The left side of the Fig. 8 shows the ROC curves of some comparison models on the BUSI dataset, from which it can be seen that the CNN-based approach overall outperforms the Transformer-based approach. We believe this is because the BUSI dataset has fewer samples, and the CNN-based methods have an advantage with less data. Comparing all the competing methods, our Eff-CTNet obtains the highest AUC value. The left side of Fig. 9 demonstrates the confusion matrix of Eff-CTNet on the BUSI dataset, from which we can see that the best classification is achieved for the normal class.

#### Results of comparison experiments on the COVID19-CT dataset

**Table 5:** Results of comparison experiments on the COVID19-CT dataset.

Method(year)	Params (M)	FLOPs (G)	Acc	F1	Precision	Recall	Auc
ResNet50 (2016)	23.5	4.1	0.8716	0.8774	0.8947	0.8608	0.8724
MobileNetV2 (2018)	2.2	0.3	0.8716	0.8805	0.8750	0.8861	0.8706
EfficientNet-B0 (2019)	4.0	0.4	0.9054	0.9079	0.9452	0.8734	0.9077
RepVGG (2021)	43.7	9.9	0.8986	0.9057	0.9000	0.9114	0.8977
ConvNext-S (2022)	49.5	8.7	0.7838	0.7922	0.8133	0.7722	0.7846
ConvMixer (2023)	47.9	49.1	0.8851	0.8957	0.8690	0.9241	0.8823
InceptionNext-S (2023)	47.1	8.4	0.8581	0.8662	0.8718	0.8608	0.8579
FasterNet (2023)	13.7	1.9	0.8784	0.8875	0.8765	0.8987	0.8769
Swin-S (2021)	48.8	8.6	0.7027	0.7609	0.6667	0.8861	0.6894
CrossViT 18 (2021)	43.3	9.0	0.7703	0.8023	0.7419	0.8734	0.7628
MoCoViT 1.0 (2022)	7.2	0.5	0.8784	0.8889	0.8675	0.9114	0.8760
BiFormer-S (2023)	56.0	9.4	0.8851	0.8903	0.9079	0.8734	0.8860
FasterViT-2 (2023)	75.2	8.9	0.8716	0.8742	0.9167	0.8354	0.8742
Flatten-pvt (2023)	24.2	3.7	0.8581	0.8591	0.9143	0.8101	0.8616
T ransxNet (2023)	25.5	4.6	0.8649	0.8701	0.8933	0.8481	0.8661
GroupMixFormer (2023)	22.1	5.1	0.9054	0.9103	0.9221	0.8987	0.9059
Eff-CTNet(Ours)	25.2	6.4	**0.9257**	**0.9317**	**0.9146**	**0.9494**	**0.9240**

Bold indicates the optimal metric values among all compared methods.

The experimental results on the COVID19-CT dataset are shown in Table 5. Our Eff-CTNet achieved 92.57% Acc, 93.17% F1, 91.46% Precision, 94.94% Recall, and 0.9240 AUC on the COVID19-CT dataset. Compared with the baseline (RepVGG), our method achieved an improvement in Acc, F1, Precision, Recall, and AUC by 2.71%, 2.60%, 1.46%, 3.80%, and 2.63%, respectively. Our method achieves a large improvement in classification performance on both BUSI and COVID-CT datasets, which further demonstrates that our method has better classification performance on small-scale datasets compared to other competing methods. At the same time, a better trade-off between classification performance and complexity is achieved.

It is worth noting that we still observe the same phenomenon from Table 5, i.e., the CNN-based method achieves better performance than the ViT-based method on the COVID19-CT dataset. This phenomenon is the same as that observed on the BUSI dataset. The reason for this is that the total number of samples in the COVID19-CT dataset is similar to the BUSI dataset, and the amount of training data is relatively small, which is also unfavorable to the ViT-based network model, preventing it from fully exploiting its optimal performance. In contrast, our Eff-CTNet is a network model based on a tandem mixture of CNN and Transformer, which is able to simultaneously take into account both local detail information and global information, effectively reducing the loss of important feature information while learning richer feature information, which to some extent reduces the need for the network to learn through a large amount of training data. The second row of Fig. 6 shows the Grad-CAM^44^ visualization of the pneumonia sample in the COVID19-CT dataset on different methods. Among them, our Eff-CTNet localizes the lesion regions on the two lung lobes very accurately, ResNet50 and FasterViT similarly focus on some of the lesion regions, but some of the methods also incorrectly focus on image boundaries that are not related to the COVID19-CT, which further reflects the reason why these methods fail to achieve a good classification performance. The middle of Fig. 7 shows the training curve of Eff-CTNet on the COVID19-CT dataset. We can also see from the figure that as the number of training epochs increases, the model gradually converges. After training for 100 epochs, the accuracy of the model changes slightly, but there is still a small improvement. In addition, we can observe that during the training process, although the overall validation loss is gradually decreasing, the fluctuations are relatively large. We analyzed that this may be due to the small number of samples in the COVID19-CT data set and insufficient data preprocessing. The ROC Curves of some of the comparison models on the COVID19-CT dataset are shown in the middle of Fig. 8, from the figure, we can see that ConvNext has the lowest AUC value among the CNN based methods. And in Transformer based method, Swin Transformer has the lowest AUC value. This is due to the fact that both the above methods require a large amount of training data to get competitive performance. Compared to other competing methods, our Eff-CTNet also shows the most competitive performance on the COVID19-CT dataset. The confusion matrix of Eff-CTNet on the COVID19-CT dataset is shown on the right side of Fig. 9.

**Table 6:** Results of comparison experiments on the Chaoyang dataset.

Method(year)	Params (M)	FLOPs (G)	Acc	F1	Precision	Recall	Auc
ResNet50 (2016)	23.5	4.1	0.8266	0.7783	0.7740	0.7849	0.8643
MobileNetV2 (2018)	2.2	0.3	0.8242	0.7702	0.7738	0.7680	0.8547
EfficientNet-B0 (2019)	4.0	0.4	0.8532	0.8021	0.8074	0.7986	0.8745
RepVGG (2021)	43.7	9.9	0.8532	0.7999	0.8043	0.7960	0.8736
ConvNext-S (2022)	49.5	8.7	0.7835	0.7189	0.7155	0.7242	0.8262
ConvMixer (2023)	47.9	49.1	0.8340	0.7736	0.7827	0.7691	0.8562
InceptionNext-S (2023)	47.1	8.4	0.8481	0.7970	0.8023	0.7925	0.8705
FasterNet (2023)	13.7	1.9	0.8439	0.7936	0.8025	0.7889	0.8682
Swin-S (2021)	48.8	8.6	0.8513	0.8029	0.8109	0.7983	0.8744
CrossViT 18 (2021)	43.3	9.0	0.8125	0.7529	0.7674	0.7436	0.8391
BiFormer-S (2023)	56.0	9.4	0.8312	0.7628	0.7860	0.7591	0.8503
FasterViT-2 (2023)	75.2	8.9	0.8320	0.7577	0.7745	0.7483	0.8454
Flatten-pvt (2023)	24.2	3.7	0.8396	0.7877	0.8035	0.7766	0.8605
TransxNet (2023)	25.5	4.6	0.8439	0.7886	0.8039	0.7786	0.8627
GroupMixFormer (2023)	22.1	5.1	0.8537	0.7971	0.8167	0.7844	0.8667
Eff-CTNet(Ours)	25.2	6.4	**0.8635**	**0.8090**	**0.8191**	**0.8012**	**0.8776**

Bold indicates the optimal metric values among all compared methods.

#### Results of comparison experiments on the Chaoyang dataset

The experimental results on the Chaoyang dataset are shown in Table 6. By observing the classification metrics of each model in the table, we can find that the ViT-based method achieves a classification performance comparable to the CNN-based method. More specifically, GroupMixFormer achieves the second Acc among all the compared methods, and Swin Transformer’s five metrics are even at the top of the list, which is completely different from the results on the two small-scale datasets of BUSI and COVID-CT above. We believe that the reason for this phenomenon is that the total number of samples in the Chaoyang dataset is about eight times the number of samples in the first two datasets, and with the increase in the amount of training data, the advantage of Swin Transformer comes out. Our Eff-CTNet also obtains state-of-the-art Acc, F1, Precision, and AUC on the Chaoyang dataset. Eff-CTNet’s Acc, F1 and Precision are improved by 1.31%, 1.50%, and 1.72%, respectively, compared to RepVGG, which is the best-performing CNN-based method. The improvement of Eff-CTNet’s classification performance on the Chaoyang dataset further validates the effectiveness and robustness of our proposed method. We show the Grad-CAM^44^ visualization results of adenocarcinoma samples under different competing methods in the third row of Fig. 6. By comparing the visualization results, our method is able to focus on the lesion area better than other competing methods. The right side of Fig. 7 shows the training curve of Eff-CTNet on the Chaoyang dataset, from the figure we can observe that the model’s validation loss and accuracy have reached convergence when training for roughly 100 epochs, while the model’s training loss and accuracy converge at roughly 150 epochs. As the number of training epochs increases, a slight overfitting phenomenon occurs. We analyze the reason for this phenomenon is that there is a lot of noise in the training data of the Chaoyang dataset^42^, and the number of samples in the four classes varies a lot, and the model overfits the noise or a certain class and ignores the real information. On the right side of Fig. 8, we show the ROC curves of different methods on the Chaoyang dataset. On the Chaoyang dataset, the different compared methods all show competitive performance, even the Transformer-based method outperforms the CNN-based method overall. This is because the number of samples in the Chaoyang dataset is much larger than that in the BUSI and COVID19-CT datasets, and thus the Transformer-based method demonstrates advanced performance. And our Eff-CTNet combines the advantages of CNN and Transformer, and thus also achieves the highest AUC value on the Chaoyang dataset. In the middle of Fig. 9, we show the confusion matrix of the proposed method on the Chaoyang dataset.

### Ablation study

**Table 7:** Ablation study of different pruning methods in ET module on three datasets.

Id	Method	Params (M)	FLOPs (G)	Acc	F1	Precision	Recall	Auc
BUSI								
(0)	Baseline	45.8	9.9	0.9133	0.8964	0.9102	0.8869	0.9162
(1)	(0)+ET Module	53.2	11.6	0.9297	0.9259	0.9295	0.9226	0.9386
(2)	(1)+EC Module	25.5	6.4	**0.9333**	**0.9261**	**0.9326**	**0.9226**	**0.9404**
COVID19-CT								
(0)	Baseline	45.8	9.9	0.8986	0.9057	0.9000	0.9114	0.8977
(1)	(0)+ET Module	53.2	11.6	0.9257	0.9308	**0.9250**	0.9367	**0.9249**
(2)	(1)+EC Module	25.5	6.4	**0.9257**	**0.9317**	0.9146	**0.9494**	0.9240
Chaoyang								
(0)	Baseline	45.8	9.9	0.8504	0.7940	0.8019	0.7887	0.8689
(1)	(0)+ET Module	53.2	11.6	0.8565	0.8066	**0.8229**	0.7952	0.8729
(2)	(1)+EC Module	25.5	6.4	**0.8635**	**0.8090**	0.8191	**0.8012**	**0.8776**

Bold indicates the optimal metric values among all compared methods.

**Contributions of different modules**: In order to assess the impact of the ET module, and the improved EC module, on the classification performance and complexity of the model, we conducted an ablation experiment. While assessing the contribution of the ET module alone, the rest of the structure of the network was kept consistent with the baseline, and then we performed an ablation experiment on the improved EC module based on the use of the ET module at each stage. The results of the ablation experiments on the BUSI, COVID19-CT, and Chaoyang datasets are shown in Table 7, respectively. By observing the experimental results in the table, we can see that adding our ET module alone on the baseline can effectively improve the classification performance on the three datasets. However, the increase in classification performance also increases the complexity of the model. To further reduce the complexity of the model, we improve the number of repetitions and channels of the EC module in the last two stages of the network. We reduced the number of repetitions of the EC module from 4,6,16,1 to 2,4,14,1 in each of the four stages, and on the basis of the above improvements, we reduced the number of channels from 512 and 1024 to 384 and 576 in stages 3 and 4. Interestingly, we found that this improvement led to another reduction in the number of parameters and FLOPs of the model, but instead, the model’s classification performance on the three datasets the classification performance of the model on the three datasets is improved. We analyze that the reasons may be twofold: 1) Eff-CTNet is a hybrid model composed of EC and ET modules interacting in tandem, and each stage of the network can learn both local and global information in medical images well, so the network does not need a large scale to learn rich feature information. 2) In this paper, we used two small-scale datasets with a small number of samples. If the network model is too large instead, it will cause overfitting. Through this ablation experiment, we found that the model has fewer parameters, fewer FLOPs, and the best classification performance on the exact three datasets when following the above design principles. Therefore we choose this design approach to implement Eff-CTNet.

**Table 8:** Ablation study of window size in GCA module.

Window size	Params (M)	FLOPs (G)	Acc	F1	Precision	Recall	Auc
BUSI							
5	25.2M	6.5G	0.9200	0.9050	0.9194	0.8948	0.9217
7	25.2M	6.4G	**0.9333**	**0.9261**	**0.9326**	**0.9226**	**0.9404**
9	25.2M	6.5G	0.9200	0.9049	0.9522	0.8730	0.9072
COVID19-CT							
5	25.2M	6.5M	0.9054	0.9054	0.9710	0.8481	0.9096
7	25.2M	6.4G	**0.9257**	**0.9317**	**0.9146**	**0.9494**	**0.9240**
9	25.2M	6.5G	0.9122	0.9182	0.9125	0.9241	0.9113
BUSI							
5	25.2M	6.5G	0.8569	0.8023	0.8113	0.7950	0.8734
7	25.2M	6.4G	**0.8635**	**0.8090**	**0.8191**	**0.8012**	**0.8776**
9	25.2M	6.5G	0.8560	0.8047	0.8122	0.7985	0.8748

Bold indicates the optimal metric values among all compared methods.

**The impact of window size in GCA module**: The ET module is the core building block of Eff-CTNet, and the core of the ET module is the GCA module. the GCA module divides the feature map into multiple groups along the channel dimension and feeds them into different self-attention headers respectively, and then divides the feature map into n windows of size m inside each header and then performs the self-attention operation respectively, this operation can effectively This operation can effectively save computational overhead. However, we observe that the value of window size m has a significant effect on the classification performance of the model. Therefore, we conducted an ablation experiment on the effect of window size on the classification performance of the model. In this ablation experiment, we only change the size of the segmentation window in the GCA module, and keep all other structures consistent with Eff-CTNet. The results of the ablation experiment on the three datasets are shown in Table 8. From the experimental results in the table, we can see that the classification performance of our method using different window sizes on all three datasets is improved to different degrees. However, when the window size is 7, Eff-CTNet achieves the optimal classification performance on all three datasets. We analyze the reason is that when the window size is 7, the size of the feature maps in each stage of our Eff-CTNet can be divided by integer, and thus the feature maps can be evenly divided into n blocks with a window size of 7. The method of integer division equalization avoids the network from mislearning or underlearning the feature information and thus obtains better classification performance.

## Conclusions

In this paper, we compare and analyze the advantages and disadvantages of CNN-based and ViT-based methods in the medical image classification task, and in response to the problems of the poor results of ViT-based methods on small medical image classification datasets with small lesion areas and the redundancy of MHSA computation in the self-attention mechanism, we propose a new hybrid medical image classification network based on CNN and Transformer for efficient hybrid medical image classification network, named Eff-CTNet. Eff-CTNet mainly consists of two basic building blocks, the EC module and ET module, stacked in tandem, which focus on local features along with global features and learn richer feature information, thus improving the performance of the network. We have conducted extensive experiments on two small-scale and one larger-scale medical image classification datasets in the public domain, and the experimental results demonstrate that our Eff-CTNet achieves more advanced performance with fewer parameters and FLOPs. At the same time, our Eff-CTNet has some limitations. Although our Eff-CTNet possesses a smaller computational spend, however, the size of the model is still larger compared to the state-of-the-art efficient CNN methods. In addition, our method achieves state-of-the-art performance on the relatively balanced dataset of the three categories in this paper, while the effect on the large-scale dataset with unbalanced samples is not clear. In future work, we will conduct further research to address the above two issues.

## Data Availability

The datasets generated and/or analysed during the current study are available in https://scholar.cu.edu.eg/?q=afahmy/pages/dataset, https://github.com/UCSD-AI4H/COVID-CT and https://bupt-ai-cz.github.io/HSA-NRL/ with corresponding permission.
